# Predictors of Deep Venous Thrombosis in Hospitalized Patients With Liver Cirrhosis in the US

**DOI:** 10.7759/cureus.23450

**Published:** 2022-03-24

**Authors:** Ahmad Abou Yassine, Mohammad Abureesh, Rachelle Hamadi, Loai Dahabra, Mohammad Alshami, Deeb Liliane

**Affiliations:** 1 Internal Medicine, Staten Island University Hospital, New York City, USA; 2 Gatsroenterology, Staten Island University Hospital, New York City, USA

**Keywords:** obesity, deep venous thrombosis, venous thromboembolism, hypoalbuminemia, liver cirrhosis

## Abstract

Background

Patients with liver cirrhosis were previously considered as anticoagulated; thus, their risk of developing venous thromboembolism (VTE) is lower. Recently, several studies showed contradicting results regarding deep venous thrombosis (DVT) occurrence in cirrhotic patients. The aim of this study is to evaluate the prevalence and risk associated with developing DVT in hospitalized cirrhotic patients in a large US population.

Methods

We queried the commercial database Explorys (IMB Inc., Armonk, New York), an aggregate of electronic health record data from 26 US healthcare systems. After excluding patients under 20 years old, a cohort of patients with a Systematized Nomenclature of Medicine - Clinical Terms of "cirrhosis of the liver" and "inpatient care" between 2015-2019 were identified, and prevalence of DVT was calculated in the exposure and the control groups. Statistical analysis for a multivariable model was performed. Factors adjusted for include gender, race, obesity, hypoalbuminemia, diabetes mellitus, viral hepatitis, and liver malignancy.

Results

Among 9,990,290 patients who were hospitalized between 2015 and 2019, 157,400 patients had a diagnosis of liver cirrhosis. The prevalence of DVT in hospitalized patients with liver cirrhosis was 3.29% compared to 3.18% in non-cirrhotic patients. Using the multivariate analysis model, DVT was inversely associated with cirrhosis in hospitalized patients [OR: 0.921; p<0.0001] compared to patients without liver cirrhosis. Predictors of developing DVT among patients with cirrhosis were non-Caucasian race, obesity (BMI>30), liver malignancy, hypoalbuminemia, and diabetes mellitus. Cirrhotic patients due to viral hepatitis were less likely to develop DVT [OR: 0.775; p<0.001] compared to non-cirrhotic patients.

Conclusion

In this database, although the prevalence of DVT in cirrhotic hospitalized patients was slightly higher than in non-cirrhotic patients (3.29% vs. 3.18%, respectively), cirrhosis as an independent factor was associated with less risk of DVT during hospitalization. This poses a question regarding DVT prophylaxis necessity in this group of patients. Further studies are needed to clarify the benefit and risks of DVT prophylaxis in cirrhotic patients.

## Introduction

Cirrhosis is defined as the histological development of regenerative nodules and severe fibrosis leading to end-stage liver disease [1]. It represents the end-stage of chronic hepatopathies, with a prevalence reaching around 1% in the US population. The most common etiologies of liver cirrhosis in the Western world are alcohol abuse, chronic hepatitis C, and nonalcoholic steatohepatitis [1,2].

Cirrhosis is a silent disease and often does not present clinical manifestation until liver damage is advanced. Patients usually seek medical attention when portal hypertension complications develop, such as ascites, spontaneous bacterial peritonitis, variceal bleeding, hepatic encephalopathy, and hepato-renal syndrome. It is well known that patients with liver cirrhosis have a bleeding tendency that usually manifests as variceal or non-variceal upper gastrointestinal bleeding, mucosal bleeding, epistaxis, menorrhagia, and easy bruisability [3]. It is estimated that up to 30% of individuals with gastric or esophageal varices will bleed during their lifetime [4].

Coagulation factors are mainly produced in the liver; hence we previously considered patients with liver damage are auto-anticoagulated due to lack of synthesis of coagulation factors with subsequent prolongation of their prothrombin time (PT) and international normalized ratio (INR). Theoretically, this should lower their risk of developing venous thromboembolism (VTE) [4,5]. However, the literature regarding this subject is still conflicting. Recent epidemiological prospective and retrospective studies have shown that the risk of deep venous thrombosis (DVT) in patients with liver cirrhosis can be high [6,7]. This could be due to an imbalance in the coagulation factors with the decreased synthesis of protein C and S and increased level of coagulation factor VIII [7,8,9,10].

Due to this conflicting data, it is difficult to address the need for DVT prophylaxis in these patients when hospitalized and whether it is safe to use anticoagulants when indicated [11,12]. In this study, we hypothesize that the risk of development of DVT in cirrhotic patients can be predicted based on patient baseline clinical characteristics. We, therefore, aimed to describe the variables associated with the development of DVT in cirrhotic hospitalized patients.

## Materials and methods

Database

Data was collected from the electronic medical record-based commercial database, Explorys (IBM Inc., Armonk, New York). Explorys is one of the largest databases available in the United States, with health information gathered from more than 26 healthcare systems throughout the nation. Explorys has de-identified data collected from electronic medical records of all participating institutions. Data is gathered, standardized, and stored in a cloud-based system. Explorys uses Systematized Nomenclature of Medicine - Clinical Terms (SNOMED-CT) for diagnosis instead of the commonly used International Classification of Diseases codes. By using the SNOMED-CT diagnosis, data can be extracted from Explorys in different groups. This data can be further subdivided into smaller groups by using another diagnosis or by applying various filters such as different demographic characteristics. As this database relies on de-identified data, it is considered Health Insurance Portability and Accountability Act compliant. Therefore, institutional review board approval is not necessary for studies published by using this database.

Study design

We performed a retrospective cohort analysis, which included all active medical records between June 2015 and June 2020. After excluding all the junior patients under the age of 20 years, the first cohort identified all patients with SNOMED-CT diagnosis of "liver cirrhosis". The second cohort included all other patients who did not have the diagnosis of "liver cirrhosis". Patients with SNOMED-CT diagnosis of "deep venous thrombosis" were extracted in both groups thereafter, and risk factors of DVT were identified. Basic demographic information, including age, sex, and race, along with other comorbid conditions, were identified in the study and control groups.

Statistical analysis

The overall prevalence of DVT in hospitalized patients with cirrhosis was calculated by dividing the number of DVT cases over the total number of hospitalized patients with cirrhosis. Similarly, the prevalence of DVT was also calculated among patients without liver cirrhosis. Prevalence was then calculated in smaller groups based on age and race. After the initial univariate analysis, a statistical multivariate model was performed using Statistical Package for Social Sciences (SPSS) version 25.0 (IBM Inc., Armonk, New York) to adjust for the known risk factors. These factors included age, sex, race, obesity, hypoalbuminemia, diabetes mellitus (DM), and liver malignancy. For all analyses, the adjusted odds ratio was calculated with 95% confidence intervals (CIs), and a two-sided p-value of less than 0.05 was considered statistically significant.

## Results

Descriptive analysis

A total of 9,990,290 hospitalized patients were included in the study. Among these subjects, we identified 157,400 patients with liver cirrhosis and 9,832,890 subjects who did not have liver cirrhosis.

As depicted in Table 1, patients in the liver cirrhosis cohort had a higher prevalence of malignancy of the liver (7% in liver cirrhosis group vs. 0.1% in non-liver cirrhosis group), DM (50% in liver cirrhosis group vs. 20% in non-liver cirrhosis group), heavy alcohol consumption (27% in liver cirrhosis group vs. 4% in non-liver cirrhosis group), and obesity (18% in liver cirrhosis group vs. 8% in non-liver cirrhosis group).

**Table 1:** Baseline characteristics of the study population

Baseline characteristics	Liver cirrhosis n(%) N= 157,400	No history of liver cirrhosis n(%) N=9,832,890	p-value
Age			
Adults between 20 and 65, seniors > 65	85,370 (54%). 72,030 (46%)	6,707,570 (68%), 3,125,320 (32%)	<0.001
Race			
Caucasian, African-American, Hispanic/Latino, Other	124,020 (79%), 21,110 (13%), 2,510 (2%), 9,760 (6%)	7,215,520 (74%), 1,409,940 (14%), 762,870 (8%), 444,560 (4%)	<0.001
Male	86.220 (55%)	4,047,240 (41%)	<0.001
Malignancy of the liver	11,800 (7%)	11,220 (0.1%)	<0.001
Diabetes mellitus (DM)	78,150 (50%)	1,929,450 (20%)	<0.001
Alcohol abuse	42,980 (27%)	365,450 (4%)	<0.001
Obesity (BMI > 30)	27,930 (18%)	794,870 (8%)	<0.001

In the univariate analysis, the prevalence of DVT was slightly higher among patients with liver cirrhosis when compared to the control group, with a prevalence of 3.29% versus 3.18% (p=0.0136).

Multivariate analysis

In the multivariate analysis, hospitalized cirrhotic patients were less likely to develop DVT when compared to the control group (OR, 0.921; CI 95%, 0.848-0.999) (p<0.001). Predictors of developing DVT were assessed for subjects with liver cirrhosis, and the results are shown in Table2.

**Table 2:** Predictors of DVT in hospitalized patients with liver cirrhosis DVT - deep venous thrombosis

Predictors	Odds ratio	95% CI	p-value
Malignancy of liver	1.475	1.210- 1.799	<0.001
Hypoalbuminemia	3.826	2.337- 6.263	<0.001
Diabetes mellitus	1.308	1.126- 1.521	<0.001
Obesity (BMI > 30)	1.622	1.389- 1.894	<0.001
Female vs. male	1.015	0.879- 1.174	NS
Non-Caucasians	1.217	1.025- 1.445	<0.001

Among hospitalized cirrhotic patients, the strongest predictor for developing DVT was hypoalbuminemia (OR, 3.826; CI 95%, 2.337-6.263), followed by obesity (BMI>30) (OR, 1.622; CI 95%, 1.389-1.894), liver malignancy (OR, 1.475; CI 95%, 1.210-1.799), diabetes mellitus (OR, 1.308; CI 95%, 1.126-1.521) and then non-Caucasian race (OR, 1.217; CI 95% 1.025-1.445). Gender was not associated with a significant difference (OR, 1.015; CI 95%, 0.879-1.174).

## Discussion

This study evaluated the occurrence of DVT in hospitalized cirrhotic patients compared to non-cirrhotic patients. To our knowledge, this study is one of the largest population-based analyses evaluating the risk of DVT in hospitalized cirrhotic patients. Using univariate analysis, we demonstrated that the prevalence of DVT was higher in hospitalized patients with cirrhosis compared to non-cirrhotic subjects (3.29% versus 3.18%, p-value=0.0136). However, after controlling for possible confounding factors in the multivariate analysis, cirrhosis was no longer associated with increased risk for DVT (OR, 0.921, p-value<0.0001). These findings reflect on the already existing conflicting data concerning the risk of thrombosis in cirrhotic patients.

Hemostasis in cirrhotic patients is complex, and several studies have suggested that this group of patients achieve a "rebalanced" hemostasis not reflected in laboratory tests [8]. Thus, the decision to consider anticoagulation in cirrhotic patients can be challenging for physicians. Traditionally, cirrhotic patients are considered to be at higher risk of bleeding secondary to being "auto-anticoagulated", but studies have shown that these patients might be at an increased risk for thrombosis [13]. Currently, there are no clear evidence-based recommendations concerning VTE prophylaxis and therapy in patients with end-stage liver disease [9].

Several studies have demonstrated that cirrhotic patients have an increased risk of thrombosis compared to non-cirrhotic patients. A large case-control study from the Netherlands [14] found a significantly higher prevalence of liver disease in patients with DVT compared to the control group (OR, 1.7; 95% CI, 1.0-2.9). In another nationwide Danish case-control study that included 99,444 patients with DVT and 496,872 control subjects, Sogaard et al. [15] showed that patients with liver disease have a substantially increased risk of DVT (relative risk of 1.74 in cirrhotic group vs. relative risk of 1.87 in the control group). Furthermore, a meta-analysis done by Ambrosino et al. showed that subjects with cirrhosis may exhibit a higher risk of DVT (OR 1.493; CI 95%, 1.266-1.762) [2].

However, other studies have shown that cirrhosis might be a protective factor against developing thrombosis. In a US population-based case-control study, Heit et al. demonstrated that the prevalence of severe liver disease was similar in patients with or without pulmonary embolism (PE) (5/625 vs. 6/625). The study further demonstrated that patients with severe liver disease were less likely to develop VTE compared to the control group (OR:0.1 CI:0.01-0.71) [16]. Another study done by Gulley et al. showed that cirrhosis was not associated with a higher risk of DVT (OR 0.87; p=0.06) and that the incidence of DVTs and PEs in cirrhotic patients was lower than that in patients with other medical illnesses such as chronic kidney disease, congestive heart failure and malignancy [6].

The coagulation system in cirrhotic patients is a rebalance of pro and anti-coagulants. The liver plays a key role in coagulation as all clotting factors except for factor VIII are synthesized by hepatocytes. Increased coagulability in cirrhotic patients appears to result from increased levels of factors VIII and Von Willebrand and lower levels of protein C, protein S, antithrombin III [8], and plasminogen [17] due to lack of their synthesis in a damaged liver. One study correlated hypercoagulability to Child-Pugh Classification, suggesting that worsening liver function may reflect an increasing prothrombotic profile in patients with liver cirrhosis. The extent of hypercoagulability in cirrhotic patients with Child-Pugh class C was similar to that observed in the plasma of patients with congenital protein C deficiency or factor V Leiden mutation [9]. On the other hand, several studies have demonstrated drivers of hypocoagulability in cirrhotic patients secondary to decrease the production of factors II, V, VII, IX, X, and XI produced in the liver [18]. Additionally, cirrhotic patients have thrombocytopenia secondary to splenic sequestration that contribute to a higher tendency to bleed.

In our study, hypoalbuminemia, as an independent risk factor, was associated with a higher risk for thrombosis in the liver cirrhosis group (OR: 3.8; 95% CI 2.337- 6.263). This was demonstrated in a previous study by Northup et al., where serum albumin was found to be an independent predictor of DVT (OR: 0.25; 95% CI 0.10-0.56) [19]. Also, hypoalbuminemia secondary to other diseases like nephrotic syndrome [20] and inflammatory bowel diseases [21] was independently associated with a higher risk of thrombosis in non-cirrhotic patients.

In addition, we showed that obesity, defined as body mass index (BMI)>30, was found to be associated with higher DVT risk in cirrhosis (OR: 1.62; 95% CI 1.38-1.89). Previous metanalyses, including eight case-control studies and one cohort study, concluded that the risk of development of VTE in obese patients was twice as high as compared to the non-obese population [22]. We also showed that the presence of liver malignancy was associated with an increased risk of DVT (OR: 1.475; 95% CI: 1.210-1.799). This is a well-established relationship where malignancy induces a thrombophilic state resulting in a higher occurrence of thrombotic events [23]. It is therefore not surprising that patients with hepatocellular carcinoma (HCC) would be at a higher risk of developing venous thrombosis. Established mechanisms of thrombosis in patients with malignancy include increased fibrinogen concentration or polymerization, thrombocytosis, and release of tissue factor-expressing extracellular vesicles [24].

The risk of DVT is highest among non-Caucasian subjects compared to Caucasians in our study. This has also been supported by many epidemiological studies where African-Americans exhibited the highest burden of thromboembolism amongst variable ethnicities [25,26]. Gender was not associated with a difference in the risk of DVT in our study population.

We recognize limitations to our study inherent to its retrospective observational nature. However, this study was strongly powered with patient data derived from a large database where more than nine million patients were included, increasing the generalizability of the study. The study design is based on obtaining SNOMED-CT codes for diagnosis or medical conditions; thus, the accuracy of our findings depends on the quality of the coding of DVT and liver disease diagnoses and comorbidities. International Classification of Disease 9 (ICD-9) and SNOMED-CT are medical terminology systems for recording medical diagnoses; however, SNOMED-CT has more concepts to be coded per clinical document than the ICD-9 [27] that makes it more accurate in terms of enlisting pertinent clinical information. Another limitation of this study was that it was unable to capture information that is unavailable in the Explorys database, such as socioeconomic status, geographic data, patient medications, or laboratory studies. Finally, some patients may have received care in facilities, which do not send their information to the Explorys database, and this may have affected the results.

## Conclusions

Our large population-based study demonstrated that after controlling for potential confounding factors, cirrhotic patients were not at an increased risk of developing DVTs compared to the control group. We also demonstrated that hypoalbuminemia, obesity, and malignancy were predictors of the development of DVT in hospitalized cirrhotic patients. Clinicians are often reluctant to prescribe anticoagulation to cirrhotic patients because of concerns regarding bleeding tendency and complications. However, increasing awareness among care providers of the potential association between these demonstrated predictors could help reduce the incidence of this preventable and costly complication. The medical literature has conflicting results on the coagulable state of cirrhotic patients; however, the previously accepted theory of cirrhotic patients being "auto-anticoagulated" is moving out of favor. This topic remains controversial, and further well-controlled prospective studies are needed. 
